# Becoming a Paralympic Champion—Analysis of the Morpho-Functional Abilities of a Disabled Female Athlete in Cross-Country Skiing over a 10-Year Period

**DOI:** 10.3390/ijerph20053909

**Published:** 2023-02-22

**Authors:** Wojciech Gawroński

**Affiliations:** 1 Department of Internal Medicine and Gerontology, Medical College, Jagiellonian University, 31-008 Kraków, Poland; wojciech.gawronski@uj.edu.pl or w.gawronski@medicinasportiva.pl; 2 Medicina Sportiva Practica, 31-553 Kraków, Poland

**Keywords:** paralympian, disabled athletes, cross-country skiers, exercise testing, VO_2max_

## Abstract

Changing medical classification into the functional one in disabled cross-country skiing means that the athlete’s predispositions and performance abilities most of all determine the final result in cross-country skiing. Thus, exercise tests have become an indispensable element of the training process. The subject of this study is to present a rare analysis of morpho-functional abilities in relation to the implementation of training workloads during the training preparation for a Paralympic champion in cross-country skiing when she was close to her maximal achievements. The study was performed to investigate abilities evaluated during laboratory tests and how they relate to performance outcomes during major tournaments. An exercise test to exhaustion on a cycle ergometer was performed three times a year on a cross-country disabled female skier over a 10-year period. The morpho-functional level which enabled the athlete to compete for gold medals in the Paralympic Games (PG) is best reflected in the results obtained by her in the tests in the period of direct preparation for the PG and confirms optimal training workloads in this time. The study showed, that the VO_2max_ level is presently the most important determinant of physical performance achieved by the examined athlete with physical disabilities. The aim of this paper is to present the level of exercise capacity of the Paralympic champion based on the analysis of the results of the tests in relation to the implementation of training workloads.

## 1. Introduction 

From the beginning, as a rehabilitation tool, sport for the disabled athlete has evolved to competitive sport at the Olympic level [1]. A real breakthrough in competition regarding cross-country skiing for the disabled took place at the beginning of the new millennium. Changing medical classification into the sport-specific functional one in disabled sports means that the athlete’s predispositions and performance abilities determine the final result [2]. The number of sporting events was limited by reducing the number of competition classes [3,4,5]. In the 2006 Paralympic Games (PG) in Turin, competition in cross-country skiing was narrowed down to three groups of participants: visually impaired (VI) athletes, standing skiers with physical disability, and athletes using a sit-ski [3]. For example, standing skiers with physical disability are grouped into the following sport classes: with lower limb impairments (LW 2, LW 3, LW 4) and upper limb impairments LW 5/7, LW 6, LW 8, and LW 9, which combined upper and lower limb impairments [6]. In the case of the athletes with those enumerated sport classes, VO_2max_ is different because of different disabilities and less muscles mass are involved during skiing. That is why Realistic Handicap Competition and Kreative Renn Ergebnis Kontrolle (RHC-KREK) was introduced in 2006 PG with regard to individual athletes in order to level out the chances of athletes with various physical disabilities competing in the same competition group [7].

The aforementioned changes led to the fact that training became more professional. During the 2002 PG preparations, training was divided into annual cycles as there were 4 years ahead to prepare the cross-country skiing Paralympic team [8]. Due to the fact that training-related overloads and, as a consequence, general overtraining [9] and injuries occurred [10,11], risks similar to those of able-bodied cross-country skiers increased. Subsequently, assessment of general health with preliminary screening, and particularly, assessment of endurance, has gained significance [12]. Hence, endurance tests have become an indispensable element of the training process, particularly in cross-country skiing for the disabled. A rational training program must be based on objective premises, while the selection of load should be compliant with the athlete’s individual endurance predispositions [13]. The literature of the subject lacks data concerning the physiological profile of the disabled skiers and, to date, little information has appearedabout performance abilities of disabled cross-country skiers. In the literature, there are no data published concerning the physiological profile of the top class athletes with physical disability, especially in the standing position (LW 2-LW 9). It seems that the scarcity of publications results mainly from the fact that there are only small groups of athletes with disabilities and various types and levels of disability who were examined, which is an obstacle in analyzing data and publishing the results. Additionally, there are rare data concerning athletes, e.g., with visual impartment (VI) and intellectual disability (ID). For example, Bernardi described research only on athletes with lower-limb dysfunctions, including sit-skiers or athletes performing winter sports other than cross-country skiing [14,15]. In turn, Bhambhani discussed physical capacity of a narrow group of skiers with various disabilities but included only one female with visual impairment who was skiing in a standing position [16]. Therefore, a review of the literature confirmed the scarcity of publications concerning the assessment of physical capacity of cross-country skiers with motor disabilities. Among the literature, there is one study on aerobic capacity of cross-country skiers, including three females, but the study is of athletes with intellectual disabilities (ID) [17].

The aim of this paper is to present the level of exercise capacity of a Paralympic champion based on the retrospective analysis of the laboratory test results in relation to the implementation of training loads.

## 2. Methods 

### 2.1. Study Participant

The study subject was a female with physically disabled upper limbs, which she lost in a post-traumatic amputation after an agricultural accident at the age of 3. She began training and participating in athletic runs at the age of 22, and at the age 23 (in the year 2000) she took up professional cross-country skiing and qualified as an athlete to sports class LW 5/7, i.e., skiing without poles. It was confirmed before the first international competition according to the Paralympic sport classification [18].

She was 29 years old when she won two gold medals in the 2006 PG and 33 years old when she won a bronze medal in the 2010 PG in Vancouver [19].

### 2.2. Health Evaluation

Every time before the physical exercise testsuntil exhaustion, a pre-participation examination (PPE) was carried out which consisted of a general medical examination and an assessment of ECG and blood and urine tests.

### 2.3. Exercise Testing

In the absence of contraindications, the exhaustion tests were performed three times a year from 2001 to 2010, i.e., before the General Preparation Phase (I), before the Specific Preparation Phase (II), and during the Competitive Phase (III) [8]. The procedure started with a general warm-up and was followed by the exercise test performed on a Monark cycle ergometer. Exercise loads starting from 60 W were increased by 30 W every two minutes until volitional exhaustion, normally occurring after 10–15 min. Before, during, and until the 3rd minute after the test, such variables as minute ventilation of the lungs (VE), oxygen consumption (VO_2_), and carbon dioxide production (VCO_2_) were registered with the use of a ergospirometer (MES system, Dymek, A., Kraków, Poland). At the same time, heart rate (HR) was monitored by a Polar-Electro device. Then, the maximal oxygen uptake (VO_2max_) and ventilatory threshold (V_T_) were calculated. The V_T_ was determined based on criteria described in the literature [20,21]. Arterialized blood was taken from an earlobe twice, i.e., before the test and 3 min after finishing the work on the ergometer. Blood lactate (La) concentration was determined using DR.LANGE LP 20 device. Body weight (BW) and the percentage of fat (%F) were measured with the use of a Tanita BF-662W scale before and after the test. In order to determine if a mechanical effect of the performed test worked, general quantity of the performed work (kJ) and achieved power (W) was calculated. The tests were always performed in the same room and with the use of the same equipment. Each time, the ergospirometer was calibrated with a standard mixture of calibration gases and the flow sensor was calibrated using a 3-litre hand calibration pump, taking into account atmospheric pressure as well as air temperature and humidity of the room where the tests were conducted. The calibration was performed under the supervision of a representative from MES, the manufacturer of the ergospirometer. The load was chosen on an ergometer which was calibrated each day before starting the exercise protocol. The cycling frequency was provided by a metronome. The described procedures guaranteed the repeatability of the test.

### 2.4. Training Data

Data from the realization of training workloads before the PG in Salt Lake City in 2002 and in Turin in 2006 were based on annual reports of subsequent coaches of the Paralympic team published by Chojnacki [8]. In turn, the realization of training loads in the 2009/2010 season is based on the reports that the athlete herself prepared according to the same scheme.

### 2.5. Analysis

In this study, selected results of exercise tests carried out in the periods directly preceding participation in the PGs are presented. During these periods, the athlete reached the highest exertional capability due to training optimization.

The data obtained from endurance tests underwent descriptive analysis. The level of VO_2max_ and blood La as well as work performed during the tests were assessed retrospectively in the context of the applied training workloads with the use of a visual inspection method and qualitative analysis. Additionally calculated were the mean, standard deviation, and coefficient of variation for observation values. 

This research was approved by the Bioethical Commission of District Medical Chamber (No. 70/KBL/OIL/2007). The athlete was fully informed of the purpose, terms, and conditions of the tests. The participant gave written informed consent in accordance with the Declaration of Helsinki before the start of the study and provided her consent to publish the reports in the future.

## 3. Results

### 3.1. Health Status

The PPE carried out periodically revealed no significant health-related contraindications to competitive sport. The athlete did not take part in only one test due to an upper respiratory tract infection in the season of 2001/2002. The athlete’s training did not cause any overload changes in particular spine sections due to asymmetry in the length of the left and right forearm stumps. 

### 3.2. Performance Abilities in Selected Seasons

During the follow-up in the years 2001–2010, a number of physical exercise tests were conducted by the athlete. The scores that were chosen for this study are characterized by morpho-functional features of the athlete that she demonstrated in her representative preparation season, i.e., directly preceding her participation in PG 2002, 2006, and 2010. 

#### 3.2.1. Season 2001/2002 before the Salt Lake City PG

The athlete took part in the laboratory physical exercise tests twice, i.e., during and after the preparation period. Anthropometric measurements performed during further tests revealed that her body weight increased and the amount of fat was between 17 and 24%, which meant a normal value for women.

The athlete’s HR on finishing Test 1 was above 190 bpm (beat per minute). The HR scores at the level of anaerobic threshold represented 84% of HR. Test 2 produced similar results. However, on this occasion the HR_max_ of the athlete was much lower and amounted to only 175 bpm. This resulted in a significant decrease in maximal oxygen intake, which in Test 1 amounted to 42.8 mL/kg/min and in Test 2 only 40.2 mL/kg/min. It was also influenced by an increase in body weight from 48.8 to 51.0 kg. Additionally, minute ventilation decreased from 84.4 to 74.9 L/min.

The work performed in this test characterized by the duration of the effort. The athlete did not enhance her performance compared to the first test. In this case, the duration of the effort was 10 min in both tests with the power output at the level of 180 W. The blood La at the time of exhaustion amounted to 12.6 mm/L in the first and 10.7 mmol/L in the second test. 

#### 3.2.2. Season 2005/2006 before the Turin PG

During the training season of 2005/2006 (Table 1), the examined athlete took part in three tests. The morphological indices in consecutive examinations showed body weight and body fat decreased by 16.3%, 13.3%, and 14.9%. Heart rate during the tests was (187, 192, 188 bpm in successive tests), which proves the athlete’s full engagement in the activity. The percentage value of HR_VT_ ranged between 83 and 89% HR_max_ VO_2max_ in two tests reached the level of 51.8/mL/kg/min, but it was lower (45.5/mL/kg/min) in Test 2.

The athlete’s minute ventilation in Test 1 (95.0 L/min) and Test 2 (99.6 L/min) was at a higher level than average and increased to the level of 107.4 L in Test 3. The duration of effort in the three tests was 14′30″, 10′, and 13′, respectively. The maximal power output achieved was 240, 210, and 240 W and at V_T_ power was 150, 165, and 150, respectively. The blood La at the time of exhaustion amounted to 10.6 mmol/L in the first test, 12.45 mmol/L in the second test, and 9.65 mmol/L in the third test. Respiratory exchange ratio (RER) in all tests was always above 1.1.

#### 3.2.3. Season 2009/2010 before the Vancouver PG

As in the previous season, the subject took part in three tests (Table 1). Compared to the results from the tests carried out 4 years before, morphological indices in subsequent tests revealed a slight increase in body mass and fat tissue which, in turn, meant a decrease in non-fat body mass.

HR in Tests 2 and 3 conducted in this period was lower than the expected level calculated with the formula “220 minus age”. The values of VO_2max_ in Test 1 were low, while in Tests 2 and 3 they reached the level of 51.3 and 53.8 mL/kg/min, which proved the subject’s high aerobic performance. The duration of effort and achieved power were the same in all the tests, i.e., 11 min and 210 W. The blood La at the time of exhaustion, which amounted to 11.7 mmol/L in the first test, decreased to 7.36 mmol/L in the third test.

### 3.3. How to Become a Paralympic Champion—Training Data in the 2005/2006 Season

In the season of peak sport achievements (2005/2006), 265 training hours were realized, where the majority of the training work (75.9%) was below at the V_T_, i.e., HR not exceeding 164 bpm, which was 85% HRmax. The remaining training loads included effort at the V_T_ and sporadically above the V_T_. There was only small fraction of the training workload performed above the VO_2max_ in a form of short accelerations lasting a few seconds each. Therefore, in general more than 75% of the training workload was performed below the V_T_ and about 25% in the range between the V_T_ and VO_2max_ [8].

**Figure 1:**
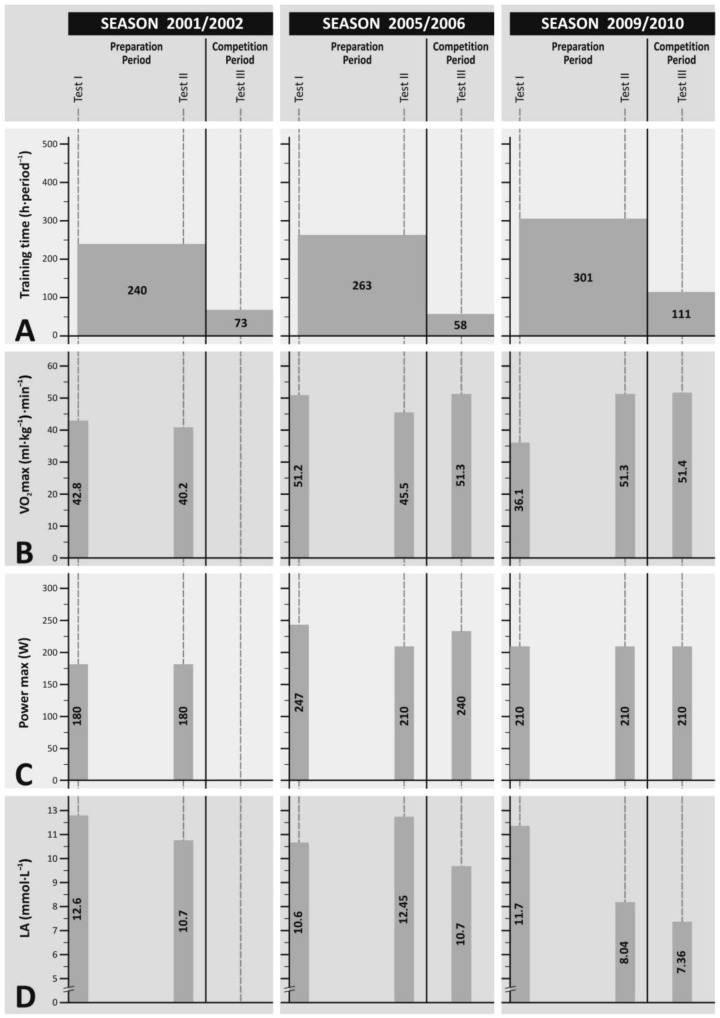
Training time represents the total amount of time spent on training in a given periods in varied seasons (2001/2002, 2005/2006, 2009/2010) and periods (Preparation Periods, Competition Period) with regard to the best results of selected variables obtained during a respective exercise tests. (**A**) Training time (h). (**B**) VO_2max_ (mL·kg^−1^·min^−1^). (**C**) Power output (W). (**D**) Blood lactate concentration (mmol·L^−1^).

A competition period is a period of training camps divided by two cycles of competitions. Afterwards, a direct preparation period for the Turin PG in 2006 started. At that time, a 10-day training camp in the high mountains was held followed by a 1-week microcycle with low training capacity. The intensity of training effort in that period at the ventilatory threshold was lowered to 50% at the cost of more intensive effort at the ventilatory threshold and above. General training capacity in the 2005/2006 season increased slightly by 3.1% compared with the 2001/2002 season [8]. In turn, in the 2009/2010 season, training capacity was by far bigger and included 414 h in total, i.e., as much as 22% more than in the 2005/2006 season. Training capacities in particular seasons in the preparation and competition periods are compared with selected results as presented in Figure 1.

### 3.4. The Morpho-Functional Abilities of the Paralympic Champion in 2006 in Cross-Country Skiing

The morpho-functional level which enabled the athlete to compete for gold medals in the Paralympic Games 2006 is best reflected in the results obtained by her in Test 3 in the period of direct preparation for the PG. In this period of top performance, the results of morphological tests were as follows: height—160 cm, body mass—51.7 kg, fat tissue—14.9%, body water—58.9%, and non-fat body mass—44 kg. In this period, the most stable body mass level, the lowest percentage of fat, and the highest non-fat body mass level were observed compared to the previous training periods. Maximal values of the selected exercise physiological variables reached by the subject in the period of 2005/2006 were as follows: heart rate—188 bpm, maximal oxygen intake—51.30 mL/kg/min, maximal minute ventilation—107.4 L/min and maximal blood lactate concentration—9.65 mmol/L with the duration of exercise—13 min. Enumerated oxygen pulse at VO_2max_ amount 14.09 mL/bt. Values at the V_T_ were as follows: heart rate—164 bpm, maximal oxygen intake—39.3 mL/kg/min, power 150 W, pedaling economy 13.54 mL/W, and in the case of VO_2_ net,12.08 mL/W.

## 4. Discussion

The longitudinal analysis of the laboratory test results in relation to the training workloads is very important for successfully facilitating potential modifications of the training process and thereby obtaining optimal performance. It is common knowledge that physical endurance, i.e., an ability to sustain long-term or hard work without signs of fatigue leading to profound systemic changes, as well as post-exertion recovery abilities, determine the performance of cross-country skiers. Physical exercise tests are used to provide information regarding a current level of endurance capabilities of athletes [22]. Exercise tolerance for disabled sports depends on a number of factors, such as metabolic profile/capacity [23], body type, and build [24,25]. The impact of these factors on endurance varies depending on the type, intensity, and duration of physical activity during cross-country competition [26]. Sports results in cross-country skiing are alsoaffected by other external factors, such as equipment, ski waxing, snow conditions, area configuration or skiing tactics, and internal factors, e.g., economy of skiing, biomechanical techniques, or anthropometric attributes [27]. Additionally, in disabled sports, a type of disability, e.g., visual disability (VI), intellectual disability (ID), or motor, muscle, and joint coordination, affects all the aforementioned factors [28]. However, the physical attributes of an athlete, particularly physical endurance, constitute the main factor. It is affected by the performance of many systems, including the cardiovascular system and the respiratory system which, to a large extent, are responsible for the aerobic potential of an athlete and especially for maximal oxygen intake [22,29]. However, there is a scarcity of studies regarding endurance capabilities of disabled athletes performing winter sports [30].

### 4.1. Maximal Oxygen Uptake

First of all, the plateauing of VO_2_- in all tests should be mentioned, which was usually visible at 30–60 s before termination of the exercise. Accordingly, the adopted observation statement classified the criterion for reachingVO_2max_ (the stabilization of oxygen uptake plateaued despite the increase of the load in the exercise and there was notable stabilization of HRmax. Additionally, RER was minimum above 1.0 or more, >1.10–1.15, and La > 8–10 mmol/L). Admittedly, plateau in oxygen consumption is the primary means of confirming that maximal oxygen uptake is attained during an exercise test to exhaustion, but it may be of crucial importance for athletes with intellectual disabilities due to a misunderstanding of test methodology [31] and the need to maintain peak effort as long as 30–60 s.

However, what causes expression of a plateau in VO_2_ at the end of incremental exercise is still unresolved. It is arguable that plateauing depends on the adopted definition and may be a primarily methodological issue and not a physiological issue. Demonstrating data may encourage the use of more objective and accurate plateau criteria and modify the current practice of using the obsolete criterion to confirm VO_2max_ [32].

The VO_2max_, as in this case, which can be compared with data from the literature [29,33] is the most significant factor in cross-country skiing. In the direct preparation period before the 2006 PG, the VO_2max_ that was achieved by the subject was 51.30 mL/kg/min (2.65 L/min). In turn, the highest level of the maximal oxygen uptake, 2.74 L/min (53.80 mL/kg/min), in the whole long-term observation period was noted during the test carried out before the 2010 PG. As a comparison, an average VO_2max_ in the test performed by three female athletes with intellectual disability was 51.8mL/kg/min [15] and theVO_2max_ reached by a female with visual impairment was 56.9 mL/kg/min [14]. Taking this into account, the maximal oxygen uptake at the level of 51.30 or 53.8 mL/kg/min may be seen as similar to other results and, according to the literature, it is a high level for a female athlete.

However, the maximal oxygen uptake achieved by the examined athlete and the above cited results of athletes with various types of disabilities differ from the VO_2max_ levels of able-bodied female skiers who obtain results at the level of 65–70 mL/kg/min and more [33]. It is worth noting that with regard to the initial tests on a cycling ergometer in 2001, the maximal oxygen uptake of the subject increased from 40.2 to 53.80 mL/kg/min. However, it should be emphasized that the studied athlete, although physically active since her trauma, started professional cross-country skiing training very late, i.e., at the age of 23, i.e., when the possibilities to improve oxygen uptake are limited for those over 30 years old [29]. However, I think the regular training compensates for the age and it is possible even after turning 100 years old [34].

Furthermore, in general, VO_2max_ during cycling exercise—as in this case—can be 10–15 % lower than during running or skiing due to the involvement of less muscle mass during cycling than during running or skiing. In the case of the athlete studied, however, VO_2max_ during cycling was probably not very different from VO_2max_ during skiing as she did not fully use her upper body during skiing due to her disability (a partial upper limbs amputation). In fact, the best measurement of oxygen uptake is the measurement taken during the field test with the use of a mobile ergospirometer. Bernardi carried out such research focusing on the biomechanics of running and training implications [15]. However, that research did not include any athlete with the dysfunction of the upper limbs, so it is hard to compare the results. In turn, other research confirmed that significantly lower levels of VO_2max_ and maximal heart rate are achieved by female and male athletes with spinal cord injuries (paraplegia, tetraplegia) competing in cross-country skiing in a sitting position [14].

For a complete analysis of endurance capabilities of the athletes, it is significant to know the values of the described indices at the ventilatory threshold. The presented results of the study participant show that in the training process, the VO_2max_ not only increased, but what is even more significant is that the anaerobic processes contributed at a higher percentage of VO_2max_ (Table 1). In general, the ventilatory threshold moved to the right, thus increasing the possibility to continue exercising without suddenly increasing fatigue. Moreover, during the peak effort, in the 2009/2010 season, decreasing concentration of La to 7.36 mmol/L in tests was noted, although time of effort (11 min), i.e., the quantity of work, did not change. It suggests a successive improvement in exercise tolerance.

### 4.2. Selected Physiological Parameters

Certainly, apart from maximal oxygen intake, other physiological indices affecting its level (e.g., HR_max_ and V_Emax_) are also significant in terms of assessing endurance capabilities of an athlete. Thus, HR_max_ of the study participant reached high values in the tests during the periods of her top performance in subsequent Paralympic seasons (191,192,189 bpm, respectively) and were higher than the age-predicted maximum HR. Similar high values of HR_max_ (192 bpm) were noted by Bhambhani in a 30-year-old athlete with visual impairment in the test [16]. Both examples confirm that the achieved VO_2max_ was at the highest possible level. However, it seems interesting that HR_max_ values noted in three young athletes (aged 17–19) with mental disabilities were at the level of 179, 178, 179 bpm which, in turn, was much lower than their age-predicted maximum HR. In this case, the achieved oxygen intake value may be seen only as VO_2peak_.

In turn, the levels of V_Emax_ in athletes with mental disabilities differed (84.9, 99.8, 120.1 L/min), while in an athlete with visual disabilities, the maximum minute ventilation reached the value of as much as 140.3 L/min [16].

### 4.3. Training Data and Analysis

Across all measures, BM, FFA, HR_max_, HR at ventilatory threshold, HR (max%), and VO_2_ (max%) were the most stable, with coefficient of variation (CV) in the 3–6% range. La at rest and effort, with CV in the 19–25% range, and fat (CV = 17%) had the highest coefficient of variation. The analysis of the presented training workloads showed a considerable domination of aerobic training, which is compliant with the recommendations resulting from subsequent tests in the years 2001–2006. Simultaneously, a considerable intensity of training and control competitions gave proper results during the first Test 1 in season 2005/2006 in the form of an increase not only in VO_2max_ by approximately 17 % but also in the ventilatory threshold level. Despite high oxygen consumption and other physiological test results, poorer results achieved during the 2010 PG (may be explained by a large increase in training capacity compared to the 2005/2006 season (Figure 1). Theoretically, these assumptions are confirmed by sports results in the following season (2010/2011), in which the athlete won a 5 km freestyle run during World Championships as well as one year later, when she received the Crystal Globe as a winner of the World Cup Series 2011/20129 [35]. Soon after this, the athlete became pregnant. As her maternity leave began 1 year prior to the next PG, she did not manage to achieve the required level of performance, which was confirmed by the control competitions. For this reason, she did not take part in the 2014 PG.

## 5. Practical Applications

The studied example shows morpho-functional capabilities (requirements to become a Paralympic champion) which should characterize an athlete competing for medals in disabled cross-country skiing during the Paralympic Games. It may be concluded that implementing a regular health assessment aimed at improving endurance capabilities of the studied athlete in 2001 was justified and innovative since, with the present level of competition in Paralympic skiing, achieving a high level of endurance and optimal health disposition during the PG is a factor determining the sports result. Thus, the laboratory exercise tests preceded by a health status evaluation are becoming an indispensable element of the training process, while the selection of loads must be based on objective factors and individual endurance predispositions of an athlete. British experiences from the Olympic and Paralympic Games in London in 2012 recommend implementing the model of combining medical care with a training process in order to achieve ethical and functional balance between medical care and optimization of sports performance [36]. This underlines the importance and the validity of the comprehensive sports and medical care system applied in Paralympic cross-country skiers. In summary, the above data provide a unique insight into the characteristics required to succeed in cross-country skiing at the PG.

## 6. Limitations and Future Research Directions

A low number of subjects, especially as described in this paper, is one of the major limitations of this study, but there is only one champion always. Additionally, other factors that may influence an athlete’s performance, such as diet, rest, supplements, and medications, were not taken into account in the study.

In the future, similar laboratory physical exercise tests should be carried out among other disabled athletes at top levels from class LW 2, LW 3, LW 4, LW 6, LW 8, and LW 9, and the obtained data should be compared. It could be useful to more objectively estimate “handicap” (RHC-KREK) for different disabilities.

## 7. Conclusions

The VO_2max_ level is presently the most important determinant of physical fitness, achieved by the examined athlete (51.3–53.8 mL/kg/min) with physical disabilities (a partial upper-limbs amputation) and is comparable with the level achieved by athletes with intellectual disabilities or visually impaired competitors which start in a standing position too.

Maximal values of the selected exercise physiological variables reached by the subject in the period of direct preparation for the PG 2006 were as follows: heart rate—188 bpm, maximal minute ventilation—107.4 L/min, and maximal blood lactate concentration—9.65 mmol/L.

The oxygen pulse at VO_2max_ amount 14.09 mL/bt. Values at the V_T_ were as follows: heart rate—164 bpm, maximal oxygen intake—39.3 mL/kg/min, power—150 W, pedaling economy—13.54 mL/W, and in the case of VO_2_ net—12.08 mL/W. 

The presented physiological, biochemical, and mechanical values show the level of endurance capabilities which characterized the female athlete who won 2 gold medals during PG 2006.

Overdose of training workloads in the season 2009/2010 was one of the reasons for a decrease in athlete performance despite the high level of VO_2max_.

Systematic medical supervision and pre-participation evaluation of a disabled athlete is very important in order to detect underlying medical problems and overtraining markers that may limit the performance or even place the athlete at “increased risk”.

The analysis of the author’s own study confirms that, in light of introduced changes in medical classification into a sport-specific functional one, the final results in cross-country skiing depend more on physical performance level than on disability type.

## Figures and Tables

**Table 1:** Morpho-functional values achieved by the study participant during endurance tests in Paralympic preparation seasons: 2001/2002, 2005/2006, 2009/2010.

Preparation Season	Tests	Bodymass (kg)	Body Fat (%)	Fat-Free Mass (kg)	HR Max (bpmin)	HR_VT_ (bpmin)	%HRmax	VO_2max_ (L/min)	VO_2max_ (mL/kg/min)	VO_2_ atV_T_ (L/kg/min)	%VO_2max_ at V_T_	VEmax (L/min)	LA (mmol/L)		Work Time (min)	Powermax. (W)	Power at V_T_ (W)
													Rest	Exercise			
2001/2002	I	48.8	20.5	38.8	191	160	81	2.06	42.2	32.3	76	84.8	0.97	12.6	10	180	120
	II	51.0	21.0	40.3	175	146	76	2.05	40.2	28.9	72	74.9	0.82	10.7	10	180	120
2005/2006	I	53.1	16.3	44.4	187	163	84	2.72	51.2	34.3	67	95.0	1.02	10.6	14.30	240	150
	II	52.7	13.3	45.7	192	171	89	2.40	45.5	34.1	75	99.6	0.69	12.45	10.30	210	165
	III	51.7	14.9	44.0	188	164	85	2.65	51.3	39.3	77	107.4	1.24	9.65	13.00	240	150
2009/2010	I	53.0	14.4	45.36	189	166	88	2.27	36.1	32.0	74	67.6	1.37	11.70	11.00	210	150
	II	52.7	19.1	42.63	184	167	88	2.71	51.3	41.7	81	95.9	0.77	8.04	11.00	210	150
	III	50.9	19.4	42.95	180	154	81	2.74	53.8	44.2	82	104.5	0.78	7.36	11.00	210	150

## Data Availability

Data Availability Statements are available in my medical office records.
